# Role of PKM2-Mediated Immunometabolic Reprogramming on Development of Cytokine Storm

**DOI:** 10.3389/fimmu.2021.748573

**Published:** 2021-10-25

**Authors:** Zhijun Liu, Yifei Le, Hang Chen, Ji Zhu, Dezhao Lu

**Affiliations:** ^1^ School of Life Science, Zhejiang Chinese Medical University, Hangzhou, China; ^2^ The Third Affiliated Hospital of Zhejiang Chinese Medical University (Zhongshan Hospital of Zhejiang Province), Hangzhou, China

**Keywords:** cytokine storm, pyruvate kinase M2, immunometabolic reprogramming, proinflammatory cytokines, inflammatory diseases

## Abstract

The cytokine storm is a marker of severity of various diseases and increased mortality. The altered metabolic profile and energy generation of immune cells affects their activation, exacerbating the cytokine storm. Currently, the emerging field of immunometabolism has highlighted the importance of specific metabolic pathways in immune regulation. The glycolytic enzyme pyruvate kinase M2 (PKM2) is a key regulator of immunometabolism and bridges metabolic and inflammatory dysfunction. This enzyme changes its conformation thus walks in different fields including metabolism and inflammation and associates with various transcription factors. This review summarizes the vital role of PKM2 in mediating immunometabolic reprogramming and its role in inducing cytokine storm, with a focus on providing references for further understanding of its pathological functions and for proposing new targets for the treatment of related diseases.

## 1 Introduction

The cytokine storm is defined as an overreaction of the immune system, which excessively activates macrophages and lymphocytes, and acutely secretes and releases high levels of proinflammatory cytokines resulting in a severe inflammatory response (1). The connections between metabolism and inflammatory response have been seen as the sally port that modulates the cytokine storm. Several novel studies have revealed the crucial role of cellular metabolism in controlling immune cell phenotype and functions. The term immunometabolism has been used to explain the intimate relationship between metabolic regulation and immune functionality. A switch of metabolic flux in immune cells from oxidative phosphorylation to aerobic glycolysis (Warburg effect) is a likely mechanism behind the sustained inflammation (2). The final rate limiting step of aerobic glycolysis is modulated by pyruvate kinase M2 (PKM2) enzyme, which been studied extensively for its important role in modulating metabolism (3). Studies report that PKM2 plays an important role in the development of some inflammatory diseases. The regulation of PKM2 can alleviate sepsis through numerous pathways (4, 5). A recent study documented that neutrophils in patients with COVID-19 exhibit immunometabolic reprogramming, with increased cytosolic PKM2, phosphorylated PKM2, hypoxia-inducible factor 1α (HIF-1α), and lactate (6). Previously, many studies demonstrated that PKM2-mediated immunometabolic reprogramming regulates the levels of cytokines through multiple pathways. The PKM2-mediated immunometabolic reprogramming promotes release of large amounts of proinflammatory cytokines, causing an excessive inflammatory response. Therefore, PKM2 is a potential therapeutic target for the treatment of cytokine storm-related inflammatory diseases. This study reviews the pathways implicated in PKM2-mediated immunometabolic reprogramming and their contribution to the cytokine storm. The findings of the study will provide a basis for the development of novel therapies for managing the cytokine storm and other inflammatory diseases.

## 2 PKM2: A Key Molecule In Modulating Inflammation in Metabolic Disorders

### 2.1 The PKM2 Metabolic Advantages Over Other Isoforms

Pyruvate kinase (PK) is a rate-limiting enzyme in glycolysis. It catalyses the transfer of a phosphate group from phosphoenolpyruvate (PEP) to adenosine diphosphate (ADP) generating pyruvate and adenosine triphosphate (ATP) (7). Pyruvate kinase exists in four isoforms including: PKL, PKR, PKM1, and PKM2. These isoforms are encoded by two PK genes, one of which encodes PKL (expressed in the liver) and PKR (expressed in erythrocytes), and the other gene encodes PKM1 and PKM2 isoforms. PKM1 and PKM2 isoforms are generated from the mutual exclusive alternative splicing of the PKM pre-mRNA, with PKM1 being formed through inclusion of exon 9, whereas PKM2 is formed through inclusion of exon 10 (8). PKM2 has low basal PK activity and is present at high levels in cancer cells, activated immune cells, and only few types of proliferating normal cells.

PKM2 occurs either as a tetramer or a dimer. The tetramer has a higher enzymatic activity and has a high level of affinity for PEP, thus can quickly catalyse PEP to form pyruvate. On the other hand, the PKM2 dimer has a lower catalytic activity and cannot produce pyruvate at a normal rate, resulting in accumulation of upstream glycolysis intermediates. These intermediates are then transferred to other pathways, such as the pentose phosphate pathway (PPP), to produce pyruvate to support biosynthesis in cells. Therefore, PKM2 is highly dimerized in cells or tissues with high nucleic acid synthesis. Studies report that PKM2 generally exists as a dimer in human monocytes and macrophages, whereas the major oligomeric status of PKM2 in murine macrophages is the monomer suggesting the existence of a species-specific regulatory mechanism (9, 10). Full activity of PKM2 requires allosteric activation by fructose 1, 6-bisphosphate (FBP) which is not the case for PKM1. The binding of FBP causes a the active site of PKM2 to combine with the ligand, therefore presenting a partially closed conformation (11). Thus, the binding of FBP remarkably increases the activity of PKM2 (12). Since the activity of PKM2 is much lower than PKM1 in the absence of FBP, proliferating cells still mainly express PKM2 but not PKM1. Thus, there exists a mechanism that can induce the transformation from PEP to pyruvate without PK. Studies have shown that PEP can be transformed into pyruvate through catalysis by phosphoglycerate mutase 1 (PGAM1), thus preventing ATP production, resulting in further inhibition of upstream steps of glycolysis (12). In addition, the PKM2 tetramer transforms into a dimer after processes, such as oxidation and acetylation, which inhibit the interaction between PKM2 and its activator FBP, thus reducing stability of the PKM2 tetramer (13). The low catalytic activity of PKM2 dimer blocks the glycolytic pathway at the last step, and the intermediates accumulate in cells. As a result, the composition of the intracellular environment becomes disordered, including accumulation of large amounts of acidic intermediates, leading to imbalance in acid-base equilibrium (14). The PKM2 dimer can enter the nucleus and bind to the transcription factors HIF-1α, Stat3 and Oct-4, thus promoting activation of the related signalling pathways and ultimately regulates biological processes including hypoxic injury and inflammation (10, 15).

Structural changes in the PKM2 tetramer deteriorate its enzymatic activity and thus mediate metabolic reprogramming, which is associated with various cellular stress responses. Upon activation, immune cells prefer glycolysis to oxidative phosphorylation to meet the urgent requirement for energy and micro-molecules. The role of PKM2 in disease pathology, such as tumour and inflammation has been extensively explored owing to the role it plays in glycometabolism and immunometabolism. Studies have shown that it plays essential roles in proliferation and transformation of tumour cells (16, 17).

### 2.2 Pharmacological Opportunities for PKM2 Modulation

This section describes activators and inhibitors under two groups including: those involved in the activation/inhibition of pyruvate kinase (PK) activity of PKM2, or those involved in isoenzyme-specific activation/inhibition of activity of PKM2. It is important to distinguish these two since they result in opposing effects (**Table 1**).

**Table 1:** Common activators and inhibitors of PKM2.

Molecules	Structural Formula	Activator/Inhibitor	Mechanism of Action
TEPP-46	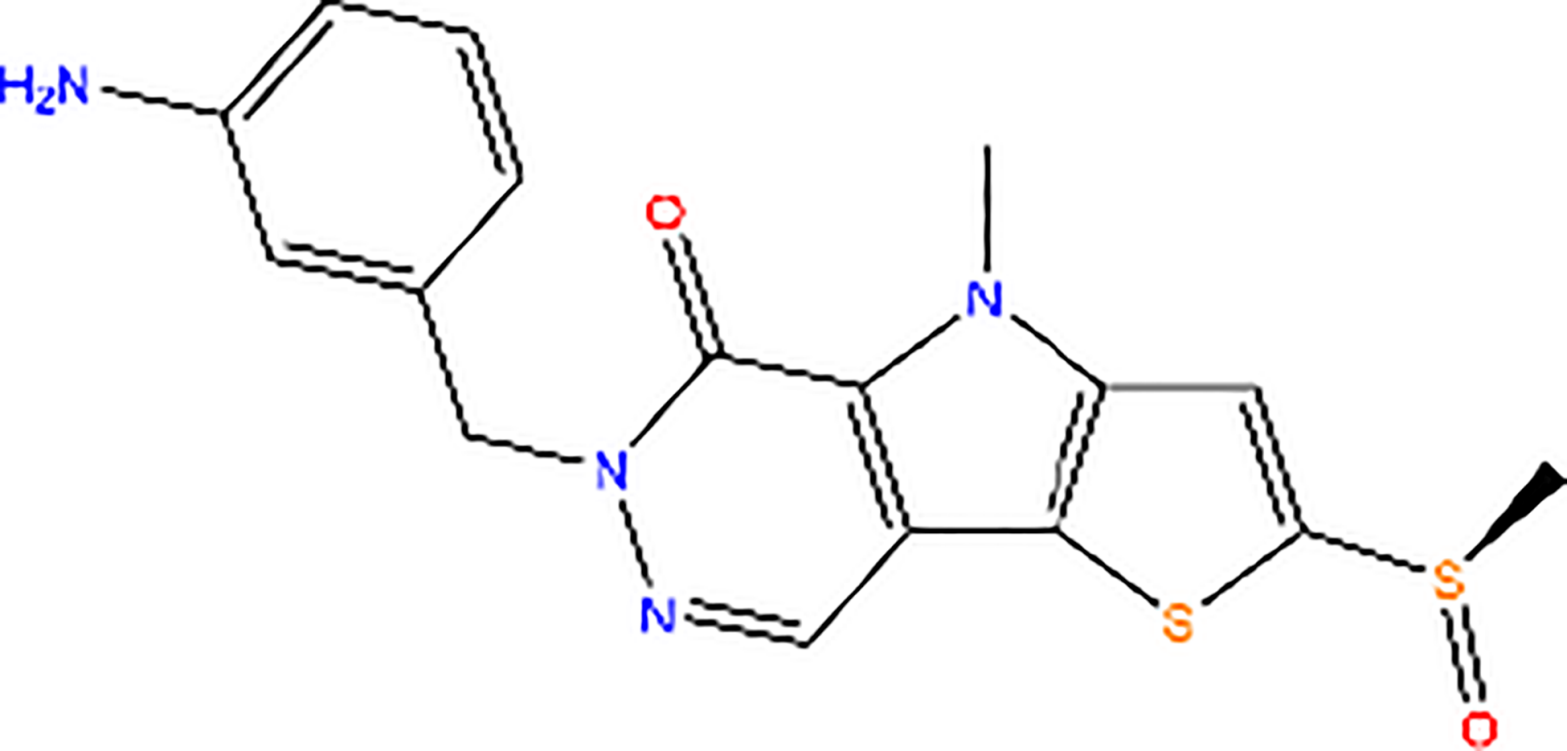	Activator of PK activity	TEPP-46 promotes PKM2 resistance to inhibition by phosphotyrosine binding
DASA-58	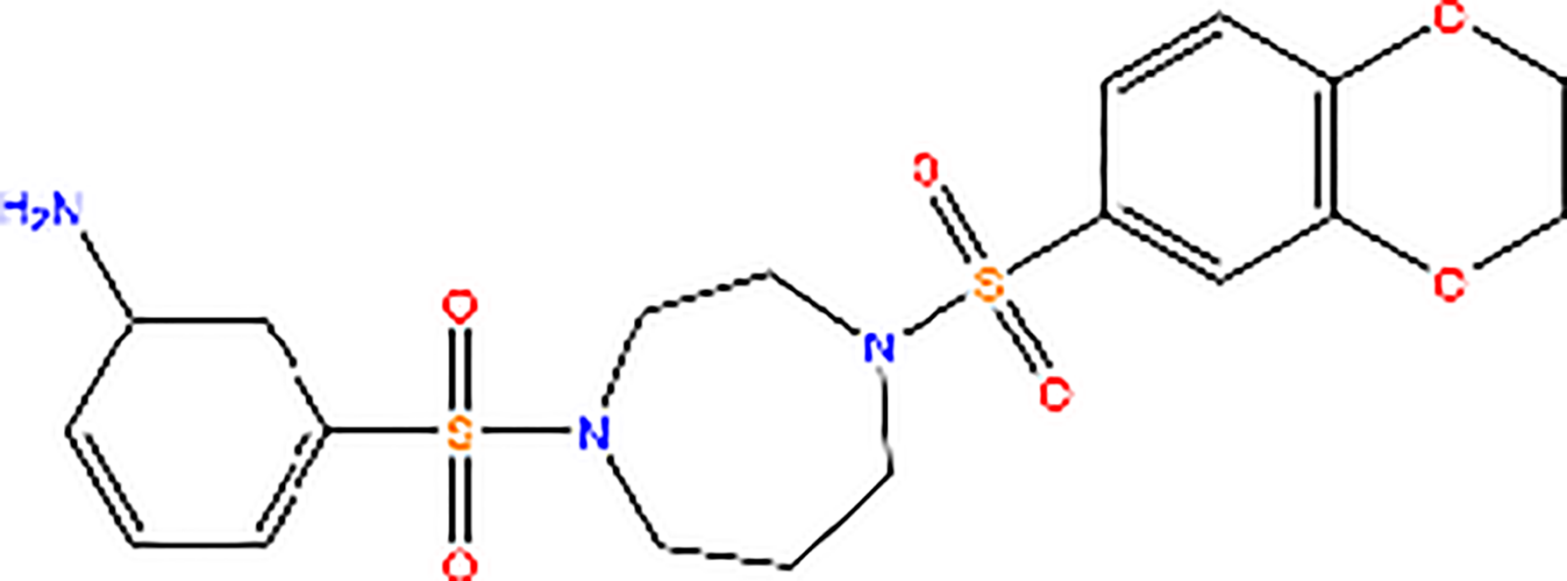	Activator of PK activity	DASA-58 promotes PKM2 resistance to inhibition by phosphotyrosine binding
SAICAR	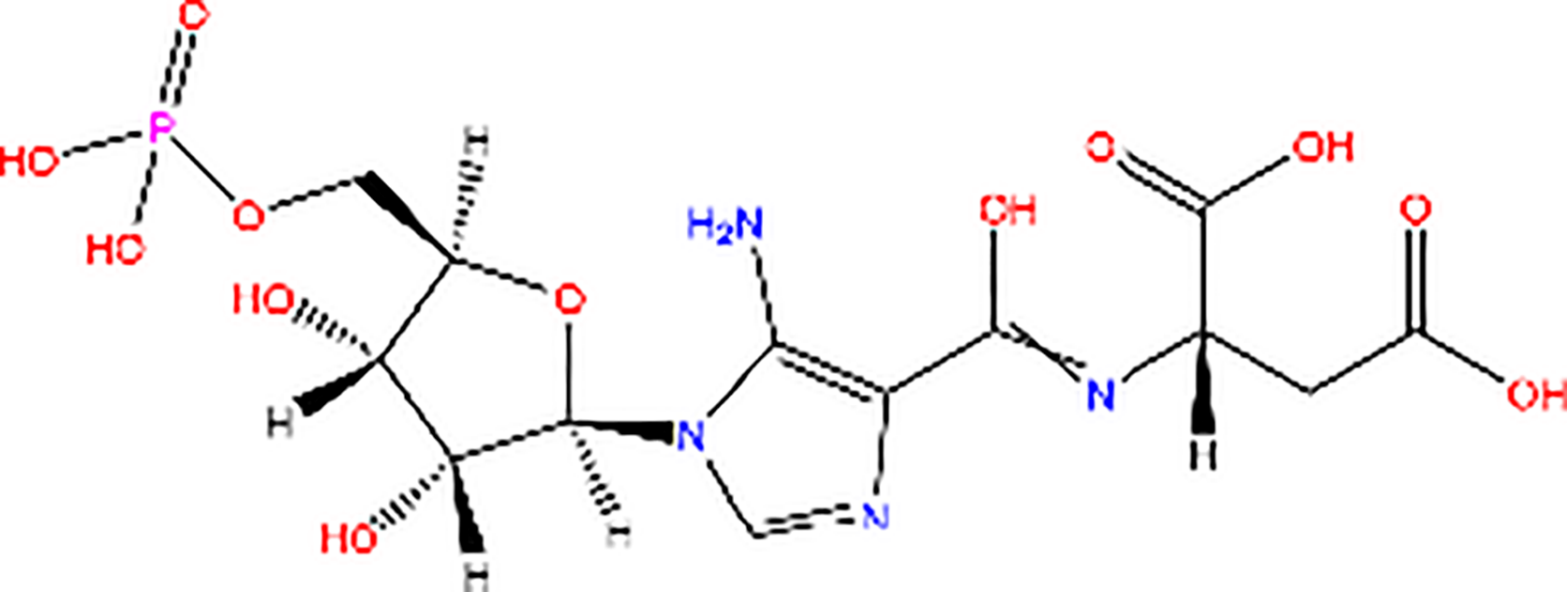	Activator of PK activity	SAICAR specifically mediates allosteric stimulation of PKM2
Compound 3K	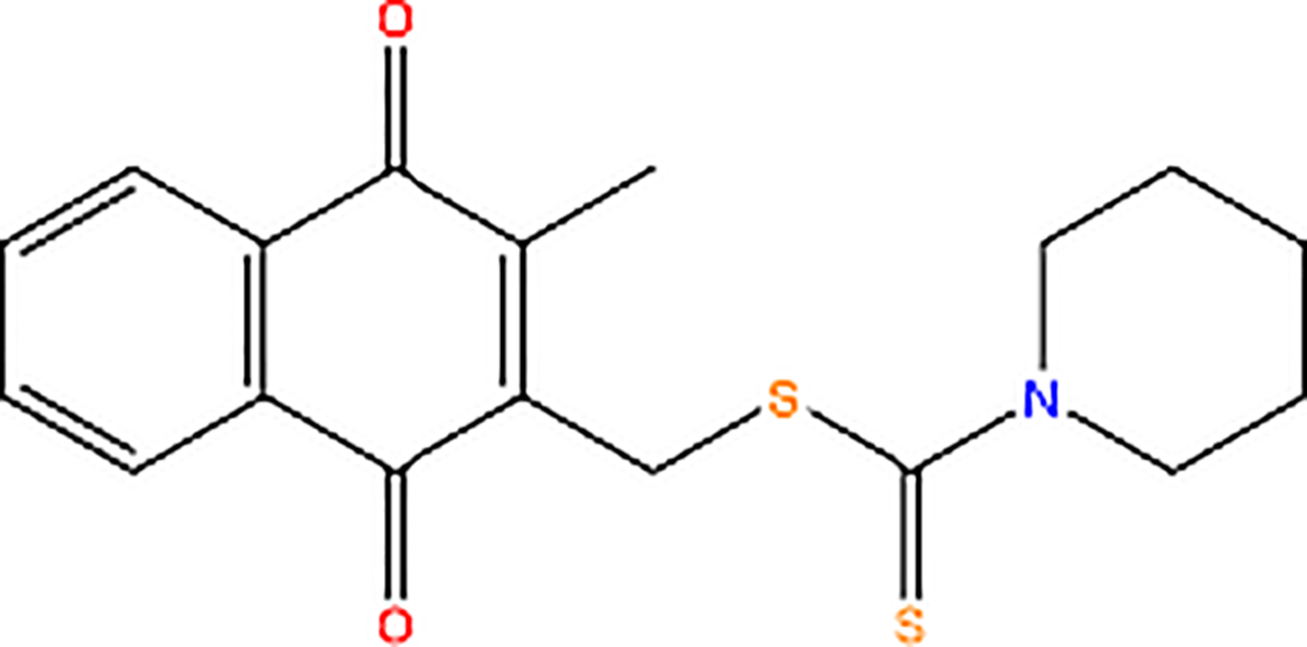	Inhibitor of PK activity	Unreported
Shikonin	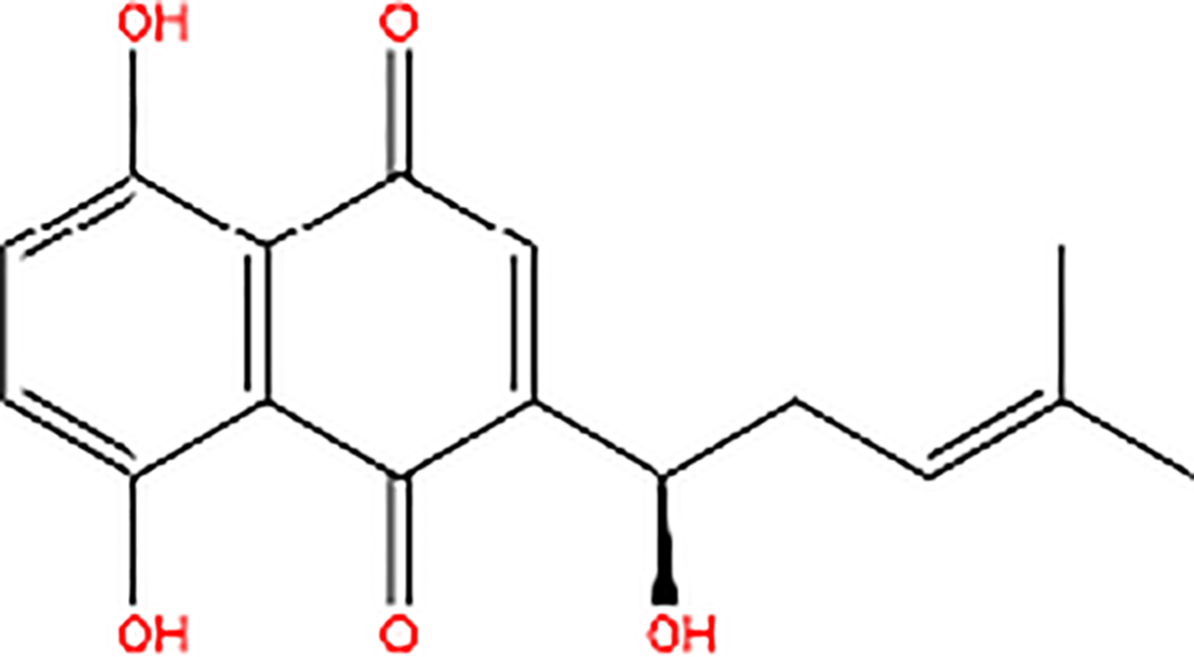	Inhibitor of PK activity	Shikonin may interact with the site responsible for allosteric regulation by FBP of PKM2
Compound 3	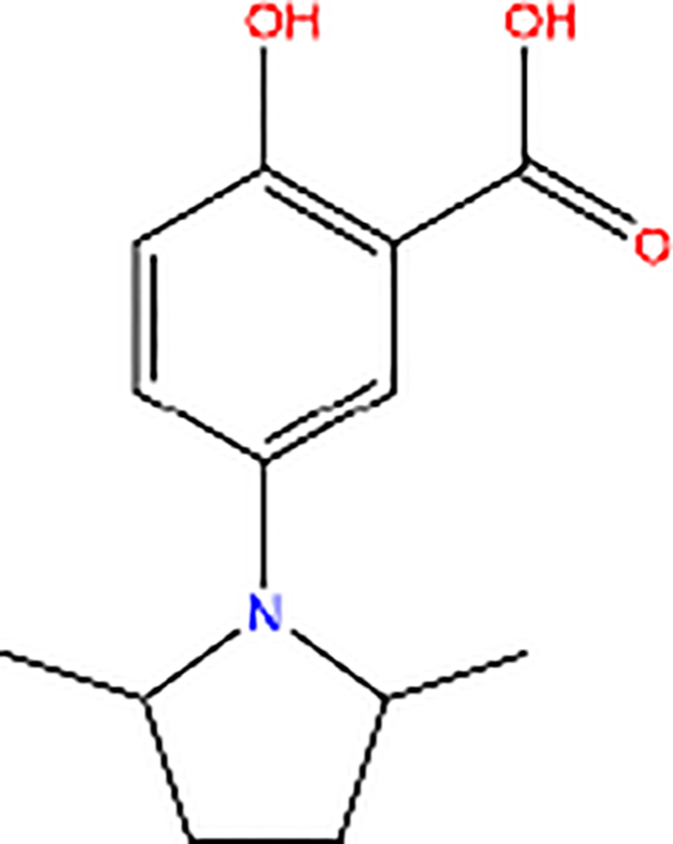	Inhibitor of PK activity	Unreported
NP-1	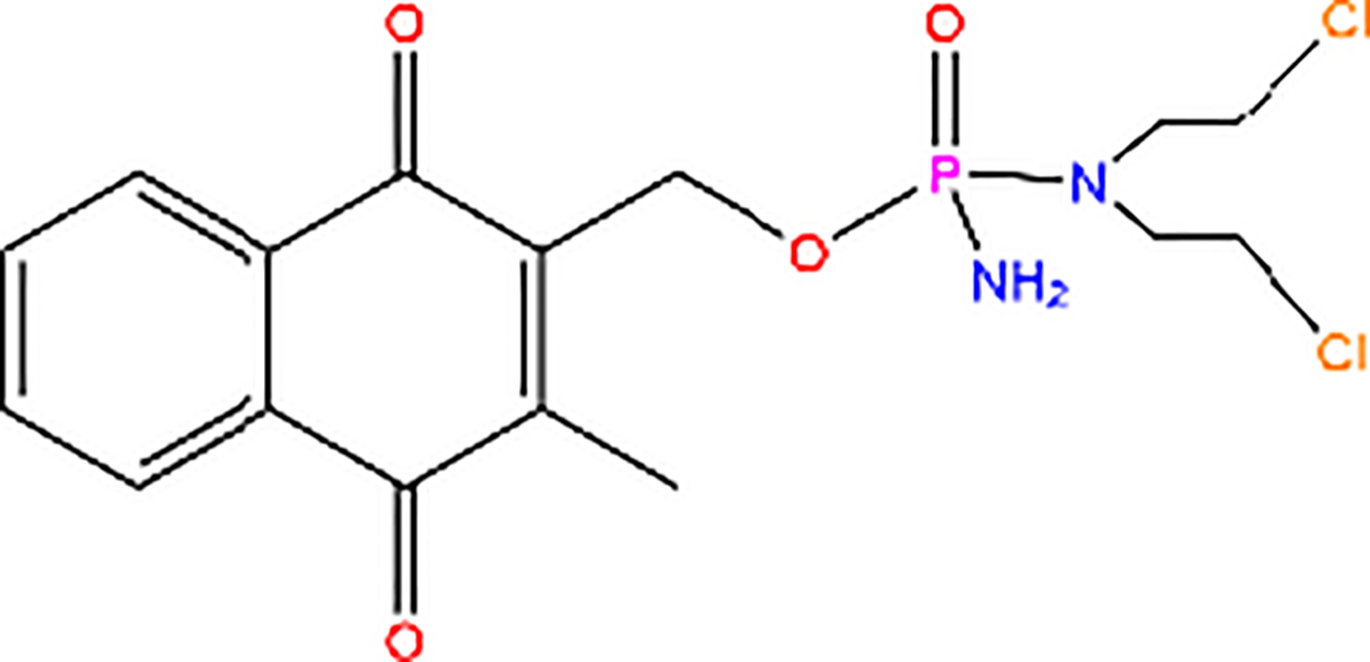	Inhibitor of PK activity	NP-1 blocks succinylation-dependent interaction of PKM2
Compound 8	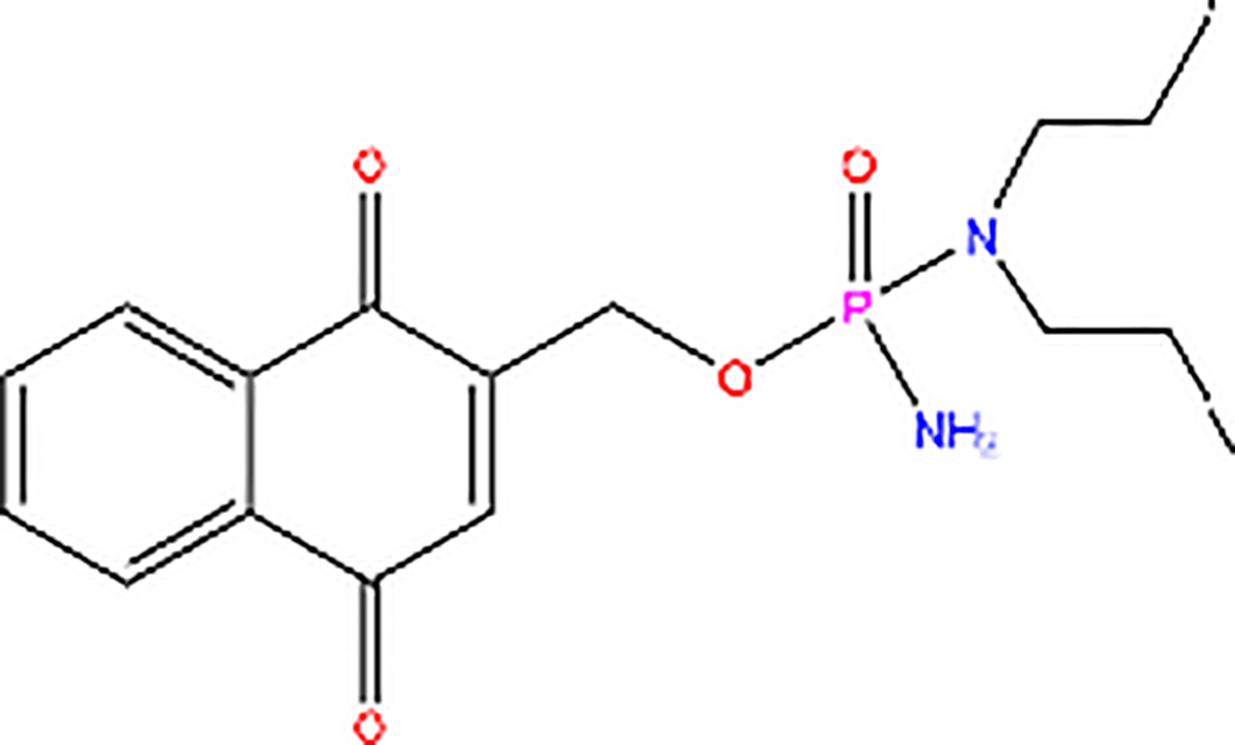	Inhibitor of PK activity	Compound 8 blocks succinylation-dependent interaction of PKM2

#### 2.2.1 Activators of PKM2

Metabolic intermediates increase the PK activity of PKM2. PKM2 differs from PKM1 by a 56-amino-acid stretch encoded by the alternatively spliced region. This stretch of amino acids forms an allosteric pocket that allows binding of FBP (11). Therefore, FBP is one of the effector molecules that activates PKM2. Studies have reported that the PKM2 tetramer can adopt the inactive T-state or active R-state conformation. After the binding of FBP, the conformation of PKM2 tetramer is changed from the T-state to the R-state, which favours the binding of PEP in the active site and enhances its enzymatic activity (18). Notably, the binding of phosphotyrosine protein can catalyse the release of FBP from PKM2 allosteric site, hence inhibiting the PKM2 enzymatic activity (13). Studies have explored a small-molecule PKM2 activator that renders PKM2 resistant to inhibition through the binding of phosphotyrosine and hinders the release of FBP from PKM2, thus increasing the PK activity of PKM2 to levels that are comparable to those of PKM1 (19). TEPP-46 binds at the dimer-dimer interface between the two subunits of PKM2 to induce the PKM2 tetramer, which is a more active state of PKM2 (20). For instance, Thieno[3,2-b]pyrrole[3,2-d]pyridazinones (21), which is a member of the TEPP-46 class is commonly used in experiments (19), and a series of substituted N,N′-diarylsulfonamides have been reported as PKM2 activators^[25]^. In addition, DASA-58, a widely used PKM2 activator, specifically activates PKM2 through similar mechanisms as TEPP-46 (19). Moreover, succinylaminoimidazolecarboxamide ribose-5′-phosphate (SAICAR), an intermediate of the *de novo* purine nucleotide synthesis pathway, specifically mediates the allosteric stimulation of PKM2 (22). These molecules activate PKM2 and play a role in latent inhibition of proliferation of tumour and development of inflammation. However, activation of PKM2 in specific subtypes of cancer cells by this class of small molecule activators results in a metabolic adaption characterized by a strict dependency on the amino acid serine for continued cell proliferation (23). In addition, other regulators that promote the post-translational modification of PKM2 can also influence its activity. Among these kinds of modifications, acetylation of PKM2 is modulated by cellular sirtuins. Sirtuins are involved in sensing nutrient availability and directing metabolic activity to match energy needs with energy production and consumption. SIRT2 (sirtuin-2) and SIRT3 (sirtuin-3) are implicated in modulation of deacetylation of PKM2, thus increasing the level of PKM2 tetramer formation and suppressing the level of PKM2 dimer, which increase the level of PKM2 activity (24, 25). SIRT6 (sirtuin-6) mediates deacetylation by binding to the nuclear PKM2 resulting in the nuclear export of PKM2, which suppresses the oncogenic functions of PKM2 (26). Notably, the effect of acetylation on PKM2 is not limited to changes in its conformation. Studies have documented that acetylation of PKM2 reduces its enzymatic activity, mainly due to its decreased affinity toward the substrate PEP, and destabilizes PKM2 through HCS70-remediated chaperone-mediated autophagy (CMA) (14). Moreover, SIRT5 (sirtuin-5) desuccinylates and activates PKM2, which plays an anti-inflammation role (27). These sirtuins can also be seen as activators of PKM2.

There are other activators that isoenzyme-specific activates PKM2, leading to an inverse effect. For instance, serine, an activator of PKM2, stimulates the activity of PKM2 but not its PK activity. Studies have shown that serine can bind PKM2 and activate it. PKM2 activity in cells is reduced when they are deprived of serine (28). This reduction in PKM2 activity results in diversion of pyruvate to the mitochondria and more glucose derived carbon is directed to serine biosynthesis to support proliferation of cells (28). The death-associated protein kinase (DAPk) which is a multi-domain serine/threonine kinase functionally increases PK activity of PKM2, thus modulating cancer cell metabolism and inhibits cell proliferation (29).

#### 2.2.2 Inhibitors of PKM2

Similarly, PKM2 has inhibitors which inhibit its activity. For example, the thyroid hormone 3,3,5-triiodo-L-thyronine (T3) and phenylalanine are the most effective enzyme inhibitors of PKM2 among more than 50 natural metabolites. T3 and phenylalanine stabilizes an inactive T-state tetrameric conformer, thus inhibiting the PK activity of PKM2 and increasing cell proliferation (30). L-cysteine amino acid induces dissociation of the PKM2 subunit, converting PKM2 from a tetramer to a dimer/monomer, thus suppressing PK activity of PKM2 (31). In addition, post-translational modifications such as phosphorylation, oxidation and acetylation can inhibit the PK activity of PKM2 (32–34).

Meanwhile, the isoenzyme-specific inhibitors that inhibit PKM2 but not its PK activity have not been reported. Isoenzyme-specific inhibitors can adjust glucose metabolism and reduce the harmful effects caused by PKM2. Compound 3K (35) and Shikonin (4, 36) are the most commonly used PKM2 inhibitors in experiments. Shikonin (SKN) is a naphthoquinone compound mainly isolated from Chinese herbal medicine *Lithospermum erythrorhizon* root and is a potent PKM2 inhibitor in cancer cells and macrophages (4, 36). Studies have reported that SKN inhibits cell proliferation and cell migration, and induces apoptosis partially through the PKM2 related pathways (37–40). However, the SKN can affect proliferation in a PKM2-independent manner, since its use may not be entirely related to PKM2 inhibition. The combination of SKN/paclitaxel overcome multidrug resistance in ovarian cancer through a PKM2-independent way of ROS generation (41). Also, whether SKN regulates other pathways through PKM2-independent ways remains unknown (37). In addition, three distinct structural classes of small molecules have been reported that exhibit selective inhibition of PKM2 over PKM1, and are the only current examples of isoenzyme-specific inhibitors (42). A small molecule referred as compound 8 can significantly reduce PKM2 activity by blocking the succinylation-dependent interaction of PKM2 and voltage-dependent anion channel proteins (VDAC)-3 thus inhibiting translocation of PKM2 into the mitochondria (43).

### 2.3 Unique Nuclear Functions of PKM2

PKM2 has unique nuclear functions which make it different from other metabolic enzymes. It contains a nuclear localization signal (NLS) encoded by exon 10. In cells activated with EGRF, PKM2 exon 10 also encodes an extracellular signal-regulated kinase (ERK) docking groove that can allow activated ERK1/2 mitogen-activated protein kinases to bind to PKM2 (44), leading to ERK1/2 phosphorylation of PKM2 at Ser37. Peptidyl-proline isomerase protein interacts with never in mitosis A (NIMA)-1 (PIN1), containing an NLS (45) and is recruited by phosphorylated PKM2 Ser37, binds to PKM2 and induces cis-trans isomerization of PKM2, which results in conversion of PKM2 from a tetramer to a dimer/monomer and then binds to importin α5. Importin α5, one of the six importin α family members (46), links NLS-containing proteins to importin β and then docks the ternary complex at the nuclear-pore complex (NPC), thus inducing translocation of PKM2 into the nucleus. In addition, a natural product micheliolide (MCL) selectively activates PKM2 through a covalent bond at residue cysteine 424 (C424) thus promoting tetramer formation, ultimately suppressing nuclear translocation of PKM2 (47). The transformation process of PKM2 is also regulated by post-translational modifications, such as acetylation and phosphorylation which shift PKM2 from tetrameric to dimeric form, leading to a higher level of nuclear transformation (48).

After localization in the nucleus, various mechanisms induce PKM2 to act as an active protein kinase. Epidermal growth factor receptor (EGFR) induces translocation of PKM2 into the nucleus through NF-κB-mediated PKM gene transcription (49, 50). Moreover, EGFR induces the transactivation of β-catenin through interaction with PKM2, which causes binding of PKM2 to Y333-phosphorylated β-catenin thus transactivating it and the protein complex subsequently binds to the cyclin D1 promoter (51). Studies have demonstrated that activation of EGFR promotes PKM2 directly by binding to histone H3 and induces phosphorylation of Thr11 on histone H3. This phosphorylation promotes dissociation of HDAC3 from cyclin D1 and MYC promoter regions, resulting in the subsequent acetylation of Lys9 on histone H3 (52). PKM2 acts as a histone kinase during PKM2-dependent histone H3 modifications and significantly upregulates the expression of c-Myc and cyclin D1. Notably, the oncogenic transcription factor c-Myc modulates different splicing of PKM1 and PKM2 by upregulating the transcription of heterogeneous nuclear ribonucleoprotein (hnRNP) proteins which bind to RNA sequences encoded by exon 9 and inhibit PKM1 mRNA splicing, leading to preferential PKM2 isoform expression (8).

HIF-1α plays an important role in metabolism reprogramming in inflammatory cells by promoting inflammatory gene expression (53). The interaction between HIF-1α and PKM2 results in a positive feedback loop. HIF-1α activates gene transcription of PKM2, whereas PKM2 interacts directly with HIF-1α subunit and promotes transactivation of HIF-1 target genes. Under hypoxic conditions, prolyl hydroxylation of HIF-1α leads to inhibition of HIF-1α proteasomal degradation, thus stabilizing and activating HIF-1α protein (54). HIF-1α dimerizes with HIF-1β in the nucleus and binds to hypoxia response element (HRE) of target genes. PKM2 contains HRE in the first intron, which allows binding of HIF-1 and directly activates its transcription (55). The PKM2 and HIF-1α complex can bind to the promoter of IL-1β (Interleukin-1β), which activates and regulates immune cells, thus playing an important role in inflammatory response (10). Moreover, hypoxia activates NF-κB, a critical component in the transcriptional pathway (56). PKM2 interacts with NF-κB subunit, p65 (57) and the interaction can trigger transcription of HIF-1α and its target genes, such as vascular endothelial growth factor (VEGF) (58). Hasan et al. reported cell-specific differences in hypoxic regulation of PKM2- HIF-1α axis. PKM2 expression is regulated by HIF-1α, however, HIF-1α expression is not regulated by PKM2 in LNCaP cells. On the contrary, PKM2 expression is not regulated by HIF-1α, but HIF-1α expression is regulated by PKM2 in PC3 cells (59). In addition, studies have reported molecules that regulate the PKM2- HIF-1α axis leading to cell metabolic reprogramming. For instance, Jumonji-C domain-containing protein 5 (JMJD5) can interact with PKM2, thus modulating nuclear translocation of PKM2 and promoting HIF-1α–mediated transactivation (60). Furthermore, the Six-Transmembrane Epithelial Antigen of the Prostate 4 (STEAP4) inhibits HIF1/PKM2 signalling (61). A combination therapy of simvastatin and sorafenib suppresses PKM2 expression and its nuclear translocation by inhibiting the interaction between PKM2 and HIF-1α/PPAR-γ (a factor that maintains energy homeostasis by modulation of glucose and lipid metabolism) (62).

Therefore, it is interesting to explore how the protein substrates bind to sites on PKM2. Studies demonstrated that the ADP binding site on PKM2 tetramer is a large hydrophobic cavity which is almost completely buried in the PKM2 tetramer structure but is accessible in the dimeric structure (11). Therefore, studies postulate that the ADP binding site can accommodate a protein substrate when PKM2 is converted to a dimer (63). Dimeric PKM2 localizes in the nucleus where it acts as a protein kinase and directly activates signal transducers and activators of transcription 3 (STAT3). However, studies have not fully elucidated whether STAT3 binds to the ADP binding site. STAT3 is a transcription activator that is activated in response to inflammatory cytokines (64). In addition, STAT3 is a unique target that can redirect inflammation to promote tumour or anti-tumour activities (65). PKM2 knockdown through intrathecal injection of siRNA has been shown to suppress chronic constriction injury (CCI)-induced STAT3 activation, thus attenuating neuropathic pain and inflammatory responses (66). Moreover, STAT3 participates in PKM2/HIF-1α feedback loop, since STAT3 activated by PKM2 can induce HIF-1α expression (67). After PKM2 transcription induced by HIF-1α, PKM2 activates STAT3 and trans-activates HIF-1α. The activated STAT3 then induces HIF-1a expression. Furthermore, cytokines, growth factors or oncogenes activate STAT3, leading to HIF-1α transcription. The STAT3/PKM2/HIF-1α feed forward loop then goes on. The function of STAT3 in promoting HIF-1α transcription has been reported. Serotonin (5-HT) phosphorylates Jak1, resulting in the phosphorylation of STAT3 which promotes ERK1/2 activation, Akt phosphorylation and HIF-1α expression (68). Although several studies report that PKM2 acts as a protein kinase, some studies oppose this theory. Hosios et al. reported that protein species labelled by [^32^P]-PEP dependent on presence of ADP but not PKM2, and PKM2-dependent transfer of phosphate from ATP directly to protein was not observed (69). This finding implies that further studies should be conducted to confirm the protein kinase function of PKM2.

**Figure 1:**
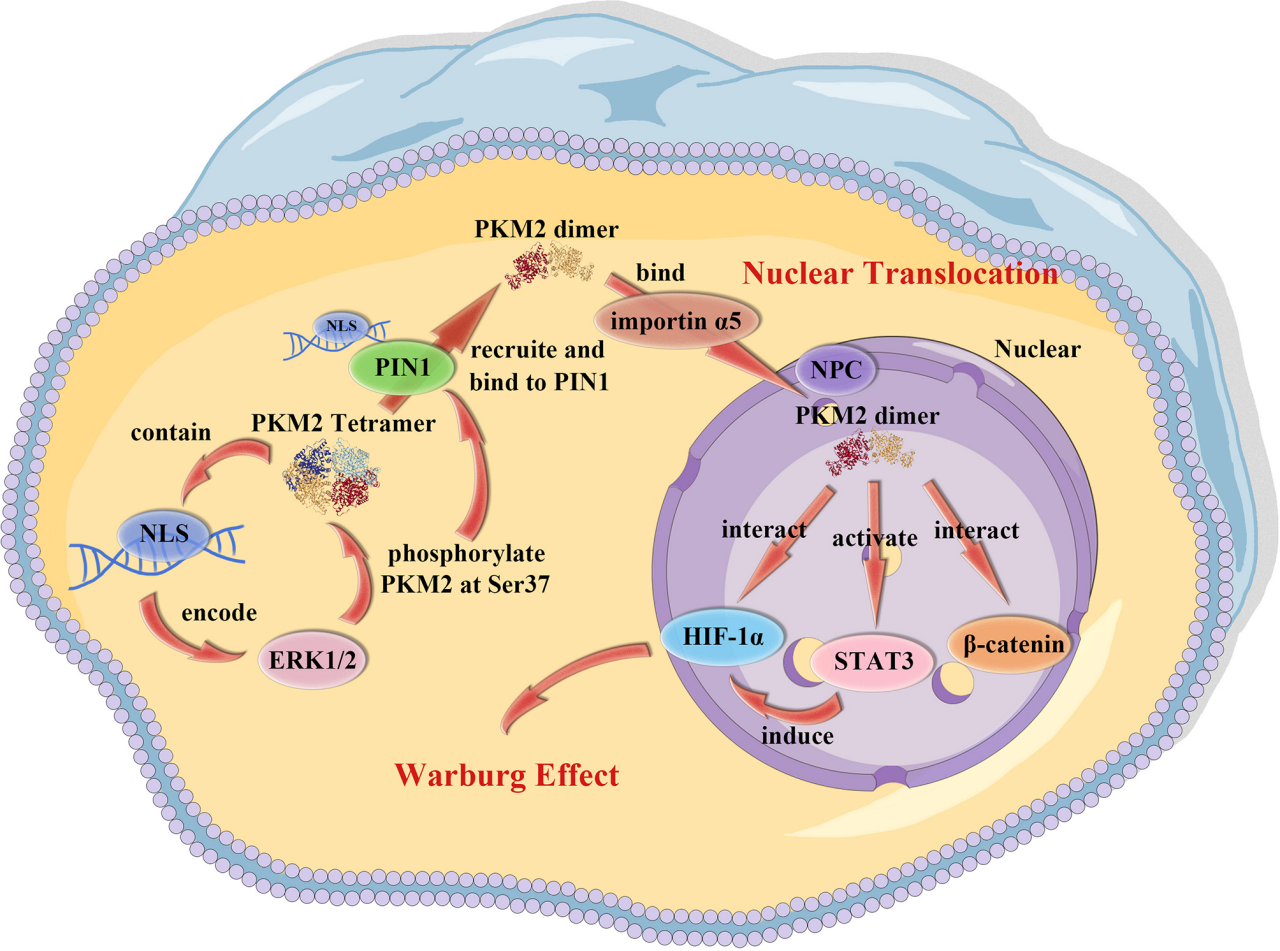
PKM2 nuclear translocation. PKM2 contains a NLS that allows ERK1/2 to bind to and phosphorylate PKM2 at Ser37. The PKM2 tetramer than recruits and binds to the NLS on PIN1, leading to the conversion of PKM2 tetramer into a dimer/monomer. The dimer/monomer PKM2 binds to importin α5 which links NLS-containing proteins to importin β and then docks the ternary complex at the NPC, inducing the translocation of PKM2 into the nucleus.

Studies report that PKM2 is a critical regulator of expression and secretion of proinflammatory mediators thus controlling transcriptional activity of HIF-1α and STAT3 pathways during inflammation (70). Therefore, PKM2 is a potential target for controlling inflammatory response (**Figure 1**).

### 2.4 Warburg Effect: A Special Metabolic Reprogramming That Can Control Immune Cell Function

In the twentieth century, a German scientist known as Warburg reported a peculiar phenomenon of metabolic reprogramming, which is currently known as the Warburg effect (71). The Warburg effect, generally seen in tumour cells or activated immune cells (2), is characterized by cell metabolism shift from oxidative phosphorylation to aerobic glycolysis under aerobic conditions. The Warburg effect was initially observed in rapidly proliferating tumour cells, however, Dr Warburg reported the same phenomenon in activated leukocytes (72). Several studies on inflammatory response report that resting immune cells derive most of their ATP from oxidative phosphorylation, whereas activated immune cells derive most of their ATP from aerobic glycolysis. The activation of immune cells, such as T Lymphocytes occurs through a process that shifts from a relative quiescence state to rapid proliferative expansion, which is regulated by signals delivered through cytokine and antigen receptors (73–76). Therefore, controlling metabolic reprogramming can enhance or suppress specific immune cell functions.

Previous studies have shown that a phenomenon like Warburg effect reported in tumours occurs during inflammatory response of immune cells. In this case, glucose metabolism in an aerobic environment is highly dependent on glycolysis and not oxidative phosphorylation (this has also been reported in some cells undergoing an inflammatory response). Reprogramming of macrophage glucose metabolism under inflammation can lead to high dependence of cellular energy metabolism on glycolysis to meet the high demand of metabolites to fight infection in an inflammatory environment. LPS induces a metabolic shift towards aerobic glycolysis in macrophages, thus promoting transcriptional expression and release of early pro-inflammatory cytokines, such as IL-1β. This is manifested by accumulation of glycolytic intermediates, rapid low-level production of ATP and accumulation of lactic acid, as well as diversion of metabolites to the pentose phosphate pathway. Therefore, the citric acid cycle (TCA) is inhibited. In addition, the onset of aerobic glycolysis increases glucose uptake and promotes the glutamine-dependent back-supplementation effect to meet succinate requirement owing to inhibition of the TCA cycle. Succinate results in succinylation of malate dehydrogenase which blocks the breakdown of downstream products of succinate and ultimately promotes accumulation of succinate, an intermediate of the TCA cycle. Abnormal accumulation of succinate in the cytoplasm creates a state of cellular ‘pseudo-hypoxia’, thus stabilizing hypoxia-inducible factor-1α (HIF-1α) by inhibiting prolyl hydroxylase. HIF-1α then enters the nucleus and binds to various regulatory factors that inversely activate transcription of glycolytic enzyme-related genes and secretion of pro-inflammatory cytokines, thus maintaining high levels of cellular glycolysis and inflammation. PKM2 is an important molecule in mediating the Warburg effect (77). PKM2 catalyses conversion of PEP in the presence of ADP to form pyruvate and ATP, the final step of the glycolytic pathway reaction. The tetrameric form of PKM2 has a high affinity for its substrate phosphoenolpyruvate (PEP) and, therefore, PEP can be converted to pyruvate at a much faster rate. However, when the PKM2 protein undergoes acetylation, phosphorylation, succinylation and other post-translational modifications, it is converted from a tetramer to a dimer/monomer. The dimeric/monomeric form of pyruvate kinase has low activity; therefore, it does not produce pyruvate efficiently and rapidly, leading to accumulation of glycolytic intermediates. Therefore, metabolism shifts to the lactate pathway to meet the high demand for ATP, leading to disruption of the TCA cycle and activation of HIF-1α. PKM2 dimers/monomers translocate to the nucleus and activate inflammation-related target genes. In the nucleus, the monomeric/dimeric form of PKM2 interacts with HIF-1α and binds directly to the IL-1β promoter. Moreover, PKM2 and HIF-1α complex stimulates polarization of M1 macrophages. On the contrary, stabilization and regeneration of PKM2 tetramers prevents accumulation of glycolytic intermediates and succinate, thus HIF-1α is downregulated. In addition, PKM2 tetramers prevent PKM2 nuclear translocation, thus reducing activation of HIF-1α target genes.

Tyrosine phosphorylation of PKM2 inhibits the role of PKM2, thus promoting the Warburg effect and proliferation of tumour cells (32). It can inhibit the tetramerization of PKM2 by disrupting binding of the PKM2 cofactor FBP. Atg7 is an important molecule involved in autophagy, which inhibits the Warburg effect by preventing tyrosine phosphorylation of PKM2 (78). In addition, the nuclear translocation of PKM2 modulates the Warburg effect (44). Nuclear PKM2 functions as a histone kinase and upregulates c-Myc expression, promoting expression of glycolytic enzyme genes which induce the Warburg effect in a feedback loop (49, 52). Therefore, when PKM2 shifts from a dimer to a tetramer, it can suppress PK activity and nuclear translocation of PKM2, thus promoting the Warburg effect, which leads to the activation of immune cells. Naturally occurring flavonoids such as apigenin, proanthocyanidin B2 and shikonin are effective regulators of aerobic glycolysis that modulate the expression of PKM2 (79). Some flavonoids exhibit anti-inflammatory activities (80). Furthermore, PKM2 regulates cellular metabolism by reducing intracellular utilization of glucose, thus increasing the α-ketoglutarate/citrate ratio, and promote formation of the product of glutamine-derived acetylcoenzyme A through a reductive pathway (81). PKM2 effectively promotes reductive glutamine metabolism, which is important for lymphocyte proliferation and cytokine production (82), macrophage phagocytic and secretory activities, and neutrophil bacterial killing (83). These findings show that PKM2 modulates metabolic reprogramming of cells to activate immune cells and trigger inflammatory response, implying that PKM2 can be inhibited to control inflammation.

## 3 PKM2: A Key Regulator of Immune Cells Metabolism

### 3.1 PKM2 Triggers Macrophage Polarization

Macrophages are important components of the innate immunity, and they play a key role in inflammatory responses. Macrophages activated by lipopolysaccharides (LPS) shift their core metabolism from oxidative phosphorylation to glycolysis even under aerobic conditions similar to the Warburg effect (84). Moreover, macrophages possess high plasticity and can adapt their phenotype to the microenvironment (85). Various signals, such as Toll-like receptor (TLR) ligands and Interferon (IFN)-γ can stimulate macrophages to form a proinflammatory M1 phenotype, promoting the secretion of high levels of proinflammatory cytokines, such as tumour necrosis factor-α (TNF-α), IL-6, IL-1β, and reactive nitrogen and oxygen intermediates. On the contrary, IL-4 and IL-13 polarize macrophages towards an anti-inflammatory M2 phenotype which exhibits immunoregulatory functions. M2 macrophages exhibit phagocytic activity and release anti-inflammatory factors such as IL-10, Arg-1, and promote tissue remodelling (86–88). Studies have shown that macrophage M2 polarization is modulated by glucose metabolism and activated macrophage metabolism is highly dependent on glucose. In normal cellular metabolism, pyruvate produced by glycolysis mainly enters mitochondrial tricarboxylic acid (TCA) cycle and undergoes a series of oxidative reactions to produce high levels of ATP. However, in M1 macrophages, glucose metabolism relies on increased rate of glycolysis to produce energy, and pyruvate produced does not enter the TCA cycle to participate in oxidative phosphorylation, thus it is converted to lactate by the lactate dehydrogenase (LDH) enzyme (89).

As we have mentioned before, previous studies reported that PKM2 is an important modulator of IL-1β production, macrophage polarization, glycolytic reprogramming, and Warburg metabolism in LPS-activated macrophages (10). PKM2 is up-regulated and phosphorylated in an activated macrophage, therefore, PKM2 primarily forms an enzymatically inactive dimer or monomer in LPS activated macrophages, which is translocated into the nucleus and binds to HIF-1α forming a PKM2-HIF-1α complex (55). The complex binds to the IL-1β promoter, thus induces the secretion of proinflammatory cytokines and triggers an inflammatory response. Furthermore, studies have reported that the PKM2 tetramer promotes M1 macrophage polarization, whereas the PKM2 dimer promotes M2 polarization. Annexin A5 is an activator of PKM2 tetramer and can inhibit phosphorylation of PKM2, thus promoting shift of macrophage phenotype from M1 to M2 and alleviates inflammation (90). On the contrary, HSPA12A interacts with PKM2 and induces its nuclear translocation, thus promoting M1 polarization of macrophages and secretion of proinflammatory M1cytokines (91). Moreover, class IIa histone deacetylases (HDACs) interact with PKM2 to initiate TLR-inducible aerobic glycolysis and drive inflammatory responses in M1 macrophages, resulting in immunometabolism-associated inflammatory responses in macrophages (92).

### 3.2 PKM2 Modulates Inflammatory Activity and Differentiation of CD4+ T Cells

CD4^+^ T cells represent an immune cell population which play a key role in immune responses. CD4^+^ T cells induce B cells to make antibodies, induce enhanced microbicidal activity in macrophages, recruit neutrophils, eosinophils and basophils to sites of infection and produce cytokines and chemokines thus inducing immune responses (93). However, over-activation of CD4+T cell can lead to development of immune-related pathologies, such as inflammatory diseases. Therefore, several studies have explored the mechanisms of interfering with intracellular metabolic pathways in immune cells to treat inflammatory diseases (94, 95). Activation of innate and adaptive immune cells is characterized by the Warburg effect-dominated metabolic reprogramming (96). Furthermore, both the activation and differentiation of T cells are highly associated with metabolic reprogramming through ligation and co-stimulation of T cell receptor (TCR) (97). Previous studies have shown that CD4^+^ T cells preferentially express PKM2 over PKM1. Moreover, PKM2 is highly phosphorylated in activated T cells, a modification that results in high levels of dimerization and nuclear translocation of PKM2 (98). TEPP-46 is a commonly-used activator of PKM2 tetramer, which inhibits the nuclear translocation of PKM2 in CD4^+^ T cells (97). In addition, Myc, HIF-1a, and mTORC1 are transcription factors that regulate related metabolic functions. Myc-dependent global metabolic transcriptome drives metabolic reprogramming in activated T cells. It has been documented that the acute deletion of Myc markedly inhibits activation-induced glycolysis and glutaminolysis in T cells which compromise activation-induced T cell growth and proliferation (99). mTOR signalling have a profound effect on the fate of CD4^+^ T cells as they promote Th1 and Th17 cell differentiation and participate in effector-like Th cells differentiation (100, 101). The inflammatory CD4+ T cell subsets rely on mTOR signalling and glycolytic metabolism, which also require expression of glycolytic enzymes, such as glucose transporter 1 (Glut1) (97). However, HIF-1α, the downstream target of mTOR, may play a role in limiting the effective function in T cells. In an hypoxic environment, HIF-1α stabilization in inhibits Th1 function (102). Additionally, the HIF-1α–dependent glycolytic pathway orchestrates the differentiation of TH17 and Treg cells through a metabolic pathway (103). Studies have reported that TEPP-46 indirectly inhibits Myc, HIF-1a, and mTORC1 signalling, which are key factors in T cell activation and functionality (97). Therefore, PKM2 modulates the functions of HIF-1α, mTORC1, and Myc and engagement of aerobic glycolysis in TCR-activated CD4^+^ T cells, thus controlling activation and effector functions of CD4^+^ T cell (98). Moreover, the PKM2-dependent glycolytic-lipogenic axis, which is a novel mechanism of metabolic regulation, is important for hyperhomocysteinemia (HHcy)-induced CD4^+^ T cell activation (104). Homocysteine (Hcy)-activated CD4+ T cell increased the protein expression and activity of PKM2 through the phosphatidylinositol 3-kinase/AKT/mechanistic target of rapamycin signalling pathway. Shikonin, a PKM2 inhibitor, effectively suppresses glucose metabolism and reduces the accumulation of lipids in Hcy-activated CD4^+^ T cells through PKM2-dependent metabolic suppression, ultimately inhibiting CD4^+^T cell inflammatory activation (105).

Differentiation of CD4^+^ T cells modulated by cytokines elicited from pathogen-activated cells plays a vital role in adaptive immunity and innate immune response. Naive CD4^+^ T cells have significant plasticity which is modulated by signals from cells of the innate immune system (106). CD4^+^ T cells are characterized into several subsets including T helper 1 (Th1), Th2, Th17, and induced regulatory T (iTreg) cells (93). The Th cells play important roles in adaptive immune responses as they secret cytokines and chemokines that activate and further recruit target cells. Notably, Th17 cells is a special IL-17-producing subset and was reported recently. Th17 cells play key roles during host defence reactions and pathogenesis of inflammation in autoimmune disease. Th17 cells produce IL-17 which promotes local chemokine production thus recruiting monocytes and neutrophils to sites of infection and inflammation (107). In addition, Th17 is a proinflammatory T cell and its differentiation is inversely related with development of anti-inflammatory regulatory T (Treg) cell (108). PKM2 plays an indispensable role during Th17 differentiation. Although PKM2 is not a prerequisite for metabolic reprogramming and proliferative capacity of Th17 cells, it promotes Th17 cell differentiation thus it is a critical nonmetabolic regulator of Th17 cell differentiation (109). TCR activation of T cells significantly increases PKM2 expression to support their differentiation. The phosphorylated PKM2 at Y105 formed into dimer when translocated into nuclear of Th17 cells. It interacts with and phosphorylates STAT3 at Y705 resultant of Th17 differentiation (109). In addition, STAT3 combines with other transcription factors and retinoic acid orphan receptor gamma T synergize to regulate transcription of Th17 cell–signature genes, thus promoting Th17 differentiation (107). Moreover, PKM2-mediated metabolic reprogramming contributes to Th17 differentiation. A previous study reported that Th17-inducing cytokines promote increased levels of calcium/calmodulin-dependent protein kinase IV (CaMK4) in naive T cells, leading to Th17 cell differentiation (110). CaMK4 binds to PKM2 and promotes its PK activity, which is required for Th17 differentiation as Th17 cells depend on glycolysis as a source of energy (111). Interestingly, the fate of T cell differentiation is determined by glycolytic activity and expression of glycolytic enzymes. The inhibition of glycolytic pathway by treatment of glycolytic pathway inhibitor *via* blocking hexokinase in T cells promotes T cell differentiation to form Treg cells rather than Th17 cells (103). The high expression of HIF-1a that increases glycolytic activity was confirmed to mediate T cell differentiation. Reports indicated that PKM2 dimer can stabilize and activate HIF-1a (54), while its tetramer form counteracts excessive rate of glycolysis. Thus, modulation on PKM2-HIF-1α axis promote Th17 differentiation, whereas activation of PKM2 (PKM2 tetramer) implicated the increment of Treg development (112). Therefore, inhibiting PKM2 dimerization can block Th17 differentiation and induce naive T cells to differentiate into anti-inflammatory Tregs, thus abrogating excessive inflammation and its damage to multiple organs.

### 3.3 Other Immune Cell Responses Activated by PKM2

Natural killer (NK) cells are lymphocytes implicated in both innate and adaptive immunity. NK cells contribute to immune defence by killing unhealthy cells and regulating responses of antigen-presenting cells and adaptive T cells (113). NK cells function as an important source of cytokines and chemokines after recognizing aberrant cells, thus they are involved in the secretion of large amounts of cytokines, such as TNF-α, IFN-γ and IL family of proteins to amplify inflammatory responses (114). In addition, NK cells interact with dendritic cells, macrophages, T cells and endothelial cells, thus inhibiting or exacerbating immune responses (115). Glycolysis activation plays an important role in NK cell effector functions (116). Studies have reported that PKM2 expression is significantly induced in activated NK cells (117). Furthermore, PKM2 is the dominant pyruvate kinase isoform in NK cells and exists mainly as a monomer and tetramer (117). PKM2 is not required for the transcription of HIF-1α and STAT5α target genes in NK cells and does not significantly regulate gene expression in activated NK cells. However, PKM2-regulated glycolytic metabolism and redox status can promote an optimal response of NK cells. PKM2-controlled metabolism is required for NK cell responses, and administration of TEPP-46 inhibits the release of NK cell proinflammatory cytokines, including IFN-γ and TNF-α. These findings show that PKM2 contributes to activation of NK cells and the secretion of NK cell proinflammatory cytokines through the glycolytic metabolism pathway.

Dendritic cells (DC) are bone marrow-derived leukocytes that engage in innate and adaptive immunity and shape adaptive immune responses based on peripheral cues (118). DCs are the most potent antigen presenting cells, and are implicated in expressing lymphocyte co-stimulatory molecules, secreting cytokines in lymphoid organs and governing both T cell immunity and tolerance (119, 120). Therefore, DCs can be targeted to manipulate the immune system. Activation of DC is inextricably linked to metabolic reprogramming just like other immune cells. Metabolism in activated DC switches from oxidative phosphorylation to glycolysis, with increase in glucose consumption and lactate production (121). Furthermore, upregulation of glucose uptake during DC activation is often accompanied by fatty acid synthesis and other pathways that rewire metabolic reactions toward anabolism (122). Notably, early glycolytic activation of DC is a common phenomenon regardless whether the stimulation is strong or weak. The main difference is that weakly stimulated DCs lack long-term HIF-1α-dependent glycolytic reprogramming and retain mitochondrial oxidative phosphorylation (123). Early glycolysis activation supports the migratory ability of DCs regardless of mitochondrial bioenergetics. Studies have indicated that PKM2 modulates the activation of DC cells by integrating metabolic reprograming with essential gene expression (124). The JNK-p300 signalling axis induces the detetramerization of PKM2 and its nuclear translocation which are crucial for the activation of DC cells. After translocating into the nucleus, PKM2 interacts with transcription factor c-Rel thus enhances IL12p35 transcription. Therefore, PKM2 facilitates activation of DCs by upregulating expression of essential cytokines. PKM2 is thus a potential new target for controlling the activation of DC cells.

The cooperation between T cells and B cells is a central tenet of immune function (125). B cells and T cells work synergistically in the immune defence system and present several common characteristics during activation. However, metabolic reprogramming in B cells is different from that in activated T cells and most other immune cells, in that oxidative phosphorylation and glycolysis are increased in BCR or LPS stimulated B cells. In addition, glycolysis results in accumulation of intermediates which are channelled to the pentose phosphate pathway (126). Moreover, glucose metabolism plays a key role in B cell activation (127). PKM2 expression and enzyme activity are increased in HHcy-induced B cells, thus promoting metabolic reprogramming that induce oxidative phosphorylation and glycolysis (128). Of note, shikonin restores Hcy-induced metabolic reprogramming in B cells, and further inhibits B cell activation and proliferation. Therefore, PKM2 is required to support metabolic reprogramming for B cell activation and function, whereas inhibiting PKM2 expression can limit B cell activation and promote inflammatory responses.

**Figure 2:**
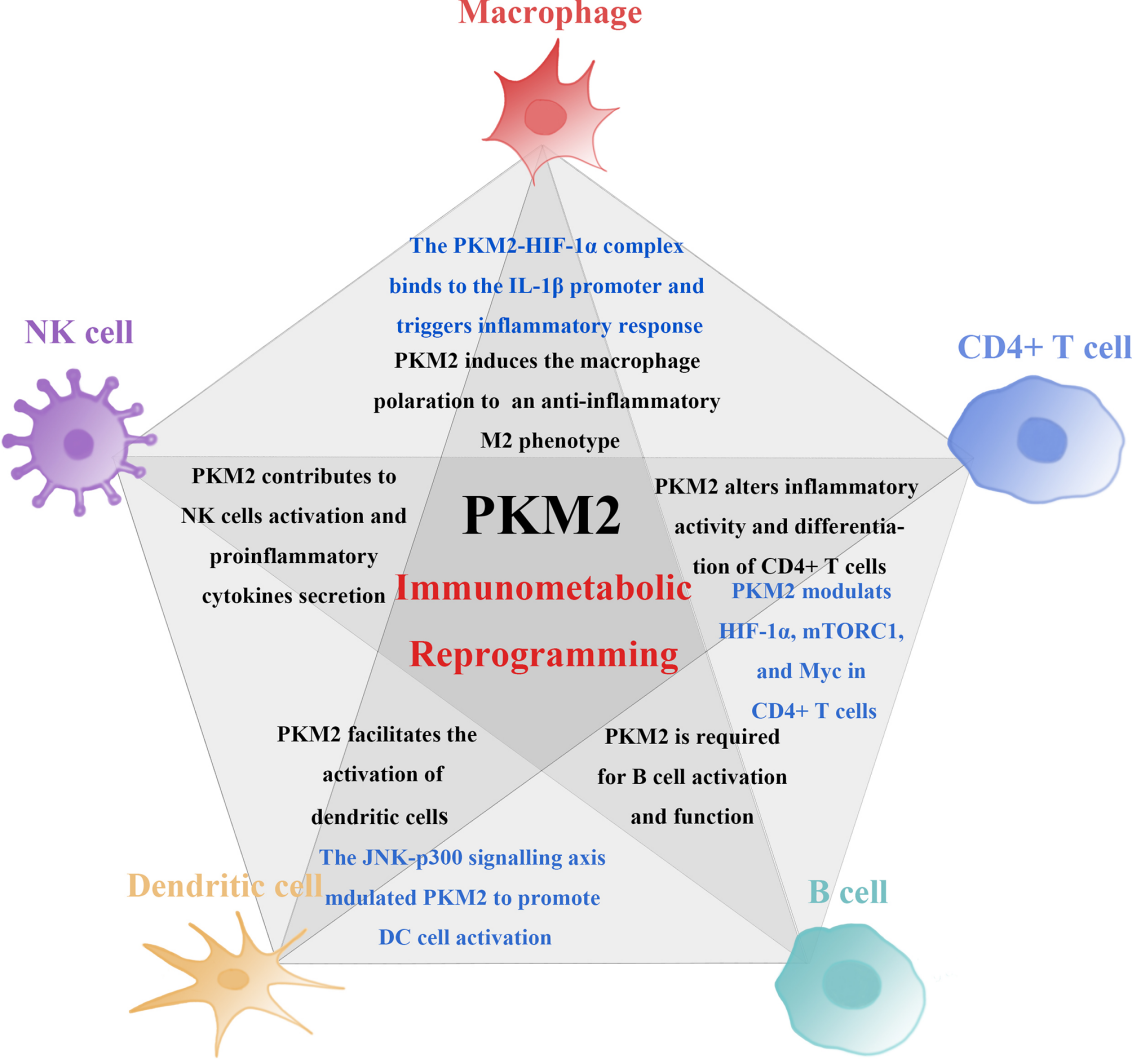
PKM2 mechanisms involved in the induction of immune cell activity and proinflammatory function. PKM2 is involved in the induction of immune cell activity and proinflammatory function. PKM2 medicates changes in the proliferation and function of immune cells through metabolic and non-metabolic pathways. PKM2-mediated immunometabolic reprogramming promotes abnormalization of immune cells. Among these immune cells, the non-metabolic pathway induced by PKM2 is in different ways (marked in blue). Firstly, PKM2 triggers macrophages to polarise from a proinflammatory M1 phenotype to an anti-inflammatory M2 phenotype. The interaction of PKM2 and HIF-1α forms a PKM2-HIF-1α complex, binding to the IL-1β promoter inducing the secretion of proinflammatory cytokines and triggers inflammatory response. Secondly, PKM2 controls the activation and effector functions of CD4+ T cell by modulating the functions of HIF-1α, mTORC1, and Myc and engagement of aerobic glycolysis in TCR-activated CD4+ T cells. Lastly, PKM2 detetramerization and nuclear translocation induced by JNK-p300 signalling axis can promote DC cell activation.

These findings show that the expression of PKM2 promotes activation of multiple immune cells by modulating both metabolic and non- metabolic pathway. Therefore, it plays a crucial role in the activation and function of immune system. Studies have reported that pharmacologically inhibiting PKM2 can control activation and proliferation of immune cells and further suppress inflammatory responses. Regulating the expression of PKM2 can thus be targeted for the treatment of inflammatory diseases through immunometabolic reprogramming (**Figure 2**).

## 4 PKM2: A Potential Promotor of Proinflammatory Non-Apoptotic-Regulated Cell Death

### 4.1 PKM2-Dependent Glycolysis Promotes Activation of Inflammasome and Induces Pyroptosis

Pyroptosis is an inflammatory form of cell death triggered by certain inflammasomes, leading to the activation of inactive cytokines like IL-18 and IL-1β and thus inducing an inflammatory response. A previous study suggested that the development of pyroptosis is closely linked to the cytokine storm (129). Inflammasomes are large multimolecular complexes containing one or more Nod-like receptors (NLRs) that control activation of the proteolytic enzyme, caspase-1 (130, 131). Caspase-1 subsequently regulates the proteolytic maturation of IL-1β and IL-18, ultimately leading to pyroptosis (132–134). Inflammasomes are assembled and activated on recognition of exogenous and endogenous stress signals released from damaged or dying cells, thus promote the pathological inflammation in sterile inflammatory diseases, such as atherosclerosis, and inflammatory diseases of acute infection, such as sepsis (135, 136). Notably, autophagy accompanies inflammasome activation to modulate inflammation by eliminating active inflammasomes (137). The canonical activation of inflammasomes modulates immune and inflammatory responses by serving as platforms for the activation of canonical caspase-1 or non-canonical caspase-11 and secretion of both early (such as IL-1β and IL-18) and late (such as HMGB1) proinflammatory mediators. Eukaryotic translation initiation factor 2 alpha kinase 2 (EIF2AK2, also known as PKR) is a protein kinase activated by viral infection and is required for inflammasome-dependent IL-1β and HMGB1 release by macrophages (138). EIF2AK2 can physically interact with inflammasomes, including NLR family pyrin domain-containing 3 (NLRP3) and Absent in melanoma 2 (AIM2). In addition, phosphorylation of EIF2AK2 is required for the activation of various inflammasomes in macrophages.

Several studies have explored pyroptosis because of its association with innate immunity and disease (139). Pyroptosis is a novel form of pro-inflammatory programmed cell death and is known as Gasdermin-mediated programmed necrotic cell death (140, 141). The pyroptosis executioner gasdermin D (GSDMD) is a substrate of both caspase-1 and caspase-11/4/5 and represents a large gasdermin family with a novel membrane pore-forming activity. Pyroptosis is associated with the activity of caspase-1, thus it causes the secretion and maturation of proinflammatory cytokines, and it is implicated in autoimmune diseases and other diseases (142–145). Although PKM2 plays a role in activating inflammasomes and inflammasome activation contributes to pyroptosis, studies have not fully confirmed whether PKM2 can mediate pyroptosis through metabolic reprogramming. Elucidating the role of PKM2 in pyroptosis can provide a basis for the development of drugs targeting PKM2. Previous studies have shown that PKM2-dependent glycolysis promotes EIF2AK2 phosphorylation thus promoting NLRP3 and AIM2 inflammasome activation in macrophages (5). The pharmacological inhibition of the PKM2–EIF2AK2 pathway inhibits release of proinflammatory cytokines thus suppressing the cytokine storm. Moreover, studies have demonstrated that PKM2-mediated-upregulation of glycolysis activates NLRP3 inflammasome leading to pyroptosis in lesioned muscle cells (146). Patients with anti‐signal recognition particle autoantibody expression present with high levels of PKM2 and IL-1β. TEPP-46 plays a protective role in the development of thoracic aortic aneurysm and dissection mediated by PKM2-induced inflammasome activation. Thoracic aortic aneurysm and dissection increases secretion of pro-inflammatory cytokines, chemokines, and reactive oxygen species (ROS) (147). These findings show that PKM2 plays a key role in inflammasome activation, and indicate that PKM2 can be pharmacologically targeted for treatment of inflammatory diseases and to alleviate cytokine storm.

Studies have indicated that stress granules which are cytoplasmic compartments enable cells to overcome various stressors. The stress granule protein, DDX3X can interact with NLRP3 to promote inflammasome activation, whereas assembly of stress granules sequesters DDX3X and inhibits NLRP3 inflammasome activation (148). Competition for DDX3X between stress granules and NLRP3 subsequent cell fate under stress conditions, determines whether the cell will die or not. Therefore, activating stress granules through the PKM2 axis to prevent inflammatory cell death is a potential therapy for inflammatory diseases. Studies have shown that hyperglycaemia increases the activation of PKM2-mediated NLRP3 inflammasomes and stress granule signalling, thus increasing plaque vulnerability and is associated with poor prognosis (149).

### 4.2 Indirect Involvement of PKM2 in Cellular Ferroptosis

Ferroptosis is new type of cell death reported recently. Ferroptosis is an iron-dependent, oxidative form of non-apoptotic cell death characterized by lipid peroxidation. Ferroptosis-inducing factors affect glutathione peroxidase (GPX) through different pathways, leading to decreased antioxidant capacity and accumulation of ROS in cells, ultimately causing oxidative cell death (150). Therefore, ferroptosis can be triggered by small molecules or conditions that inhibit glutathione biosynthesis or the glutathione-dependent antioxidant enzyme GPX4 (151). Inactivation of GPX4 induces ferroptosis by inhibiting cystine/glutamate antiporter, resulting in accumulation of ROS in the form of lipid hydroperoxides (152). Furthermore, ferroptosis is morphologically characterized by decreased or diminished mitochondria cristae, a ruptured outer mitochondrial membrane, and a condensed mitochondrial membrane (153). These morphological changes result from loss of selective permeability of the plasma membrane due to significant membrane lipid peroxidation and occurrence of oxidative stress (154). Therefore, membrane lipid peroxidation caused by ferroptosis can damage cytomembranes and promote inflammatory reactions in cells. Dysregulation of ferroptosis has been reported in multiple physiological and pathological processes, such as cancer and metabolic diseases (155, 156). In addition, studies have reported that ferroptosis plays a positive role in inflammation through immunogenicity (157). Unlike the immunologically silent apoptosis, damage-associated molecular patterns (DAMPs) and alarmins are released in ferroptosis affected cells, thus triggering cell death and promoting a series of reactions related to inflammation (158–160). Interestingly, several ferroptosis inhibitors exhibit anti-inflammatory effects.

Studies have indicated that pyruvate metabolism is associated with ferroptosis factors. Mitochondrial pyruvate carrier (MPC)-1 is an inner membrane protein that transfers pyruvate to the mitochondria (161). MPC1 expression is regulated by histone lysine demethylase 5A (KDM5A), and the KDM5A-MPC1 axis promotes cancer cell progression (162). Recent studies have shown that the suppression of MPC1 increases ferroptosis vulnerability *in vitro* and *in vivo* by promoting mesenchymal traits and glutaminolysis (163). Regulation of the KDM5A-MPC1 axis promotes cancer ferroptosis susceptibility. In addition, pyruvate kinase deficiency (PKD) causes GPX4 deficiency, resulting in mitophagy perturbation and inhibits reticulocyte maturation (164). Cytosolic L-glutamine is preferentially used as α-ketoglutarate as a source of intermediates for the tricarboxylic acid (TCA) cycle at the expense of glutathione (GSH) production, leading to GSH depletion and GPX4 deficiency. In summary, dysregulated pyruvate metabolism contributes to GPX4 deficiency resulting in oxidative imbalance. Furthermore, previous studies established a potential association between PKM2 expression and GPX4 (165). RSL3 is a small molecular compound that induces ferroptosis by inactivating GPX4. ATP level, pyruvate content and protein levels of glycolytic enzymes including PKM2 were decreased in glioma cells treated by RSL3, indicating that RSL3 induces glycolysis dysfunction, resulting in autophagic cell death (165). Therefore, the inactivation of GPX4 promotes PKM2-related glycolysis dysfunction indirectly, indicating a potential association between PKM2 and GPX4. Targeting GPX4 can regulate ferroptosis, and affects other inflammatory-related cell death. GPX4 potentially coordinates lipid peroxidation, inflammasome activation, and pyroptosis during polymicrobial infection (166, 167). Upregulation of GPX4 counter-regulates GSDMD-N mediates pyroptotic cell death in response to infectious insults, preventing lethal systemic inflammation. Therefore, GPX4 is an essential negative regulator of pyroptosis that prevents a lethal inflammatory response.

PKM2-mediated Warburg effect can induce ferroptosis. Formaldehyde induces ferroptosis by upregulating the Warburg effect in hippocampal neuronal cells (168). Analysis of upregulation of PKM2 shows that formaldehyde upregulates the Warburg effect in hippocampal tissue, and increases the level of lipid ROS and iron content. In addition, inhibition of the Warburg effect by dichloroacetate (DCA) protects hippocampal neuronal cells against ferroptosis and cell death (168). Therefore, regulation of PKM2 to suppress the Warburg effect is a feasible method for modulating ferroptosis.

**Figure 3:**
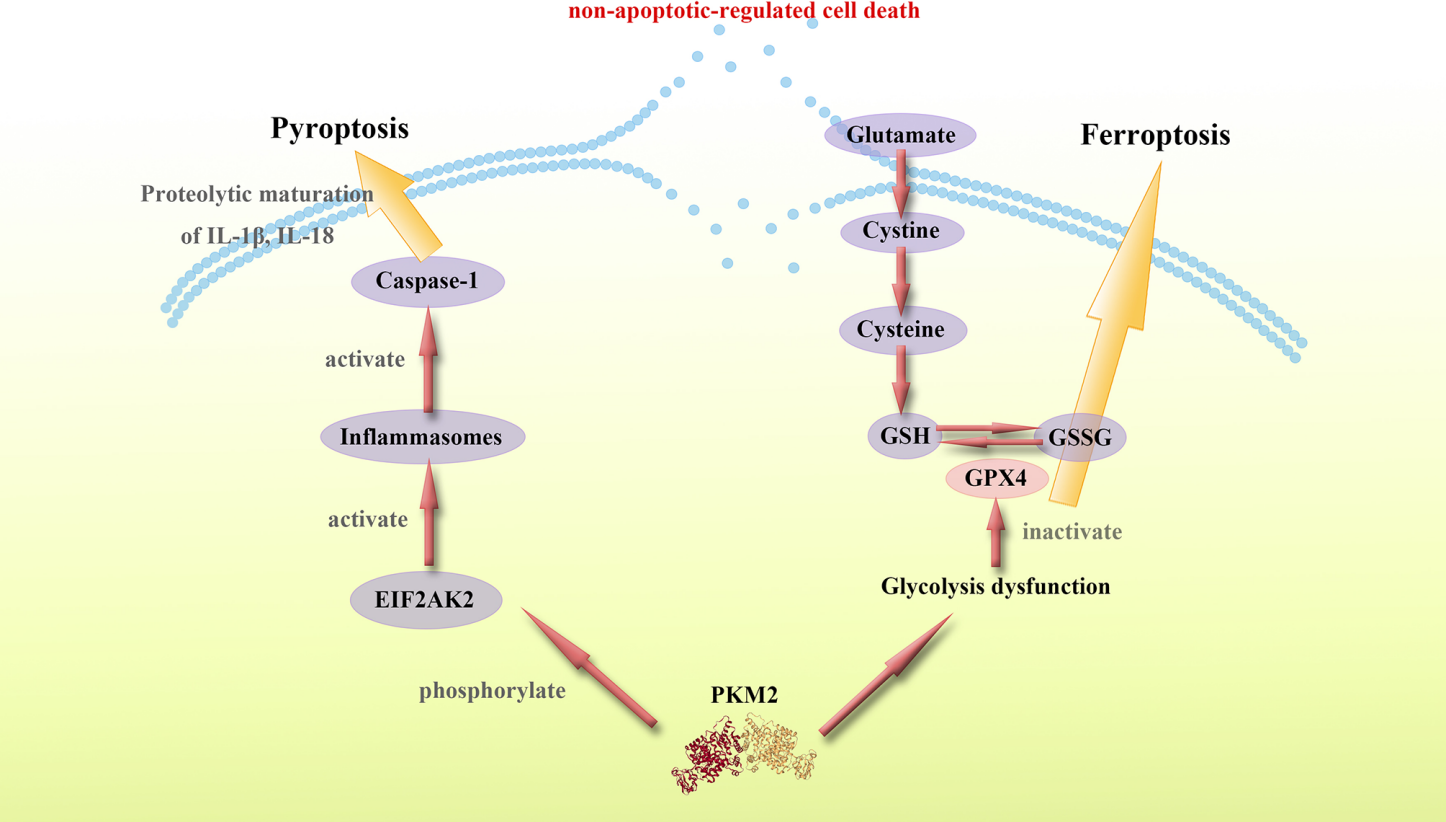
PKM2 mediates proinflammatory non-apoptotic-regulated cell death. PKM2 mediates proinflammatory non-apoptotic-regulated cell death such as pyroptosis and ferroptosis. The specific mechanisms by which PKM2 regulates pyroptosis and ferroptosis remain unknown, however, a considerable number of studies imply a potential connection between PKM2 and these non-apoptotic-regulated cell death. PKM2-dependent glycolysis promotes EIF2AK2 phosphorylation and inflammasome activation, while the activation of inflammasome also activates Caspase-1 and thus leading to pyroptosis. Moreover, PKM2-mediated Warburg effect can induce ferroptosis. The glucose metabolism dysfunction caused by PKM2 mediates the reducibility function of GSH, leading to ferroptosis. Thus, targeting PKM2 is a potential approach of regulating proinflammatory non-apoptotic-regulated cell death.

These findings show that PKM2 and PKM2-mediated metabolic reprogramming modulates proinflammatory non-apoptotic-regulated forms of cell death, such as pyroptosis and ferroptosis through multiple pathways. PKM2 can promote the secretion of proinflammatory cytokines and cause systemic inflammation. Therefore, studies should design molecules targeting PKM2 to regulate the inflammatory forms of cell death, thus suppressing inflammation and inhibiting cytokine storm (**Figure 3**).

## 5 PKM2: A Potential Therapeutic Target of Oxidative Stress and Inflammatory Damage

Oxidative stress is defined as the imbalance between the production of ROS and the endogenous antioxidant defence system. Oxidative stress has been viewed as one of the potential common aetiologies of many inflammatory diseases (169, 170). Studies have reported that dysregulated fatty acid metabolism is related to oxidative stress from the mitochondria, which drives chronic inflammation (171). Therefore, the relationship between oxidative stress and immunometabolism should be explored.

### 5.1 PKM2 Directly Induces Release of Proinflammatory Cytokines

Cytokines are key modulators of immunity. Cytokines are involved in every facet of immunity and inflammation, including innate immunity, antigen presentation, bone marrow differentiation, cellular recruitment and activation, and expression of adhesion molecules (172). High levels of proinflammatory cytokines and hyperactivation of immune cells leads to a life-threatening systemic inflammatory syndrome, which is also known as cytokine storm (1). Several studies have shown that PKM2-mediated immunometabolic reprogramming promotes the secretion of proinflammatory cytokines. PKM2 forms a complex with HIF-1α in LPS-stimulated macrophages then the complex directly binds to the IL-1β promoter, and can be inhibited by PKM2 activators such as DASA-58 and TEPP-46 (10). The activation of PKM2 by both compounds inhibits glycolytic reprogramming and succinate production. In addition, activation of PKM2 by TEPP-46 *in vivo* blocks IL-1β production induced by LPS and Salmonella, whereas production of the anti-inflammatory IL-10 is increased. PKM2 mediates production of early proinflammatory mediators, and promotes secretion of late proinflammatory mediators. PKM2 interacts with HIF-1α and activates HIF-1α-dependent transcription of enzymes involved in the Warburg effect in macrophages. PKM2-mediated Warburg effect then regulates the release of HMGB1 (4). PKM2 knockdown and pharmacological inhibition reduces serum lactate and HMGB1 levels. Shikonin protects mice from lethal endotoxemia and sepsis and other inflammatory diseases by inhibiting PKM2 expression. In addition, studies have shown that the levels of proinflammatory cytokine in injured lungs induced by mechanical ventilation significantly increased whereas CXCL14 level decreased and PKM2 expression was increased (173). During this process, overexpression of CXCL14 can alleviate ventilator-induced lung injury and inhibition of pulmonary inflammation through downregulation of PKM2-mediated cytokine production. Previous studies have reported that shikonin, metformin, vitamin K (VK)3/5 exhibit significant inhibitory effects on PKM2 expression. Moreover, these agents exhibit an anti-inflammation effect that protects lungs against sepsis-associated lung injury (174–176). Therefore, novel agents targeting PKM2 can be explored to inhibit proinflammatory cytokine production and alleviate organ failure caused by the cytokine storm. Furthermore, PKM2 plays an important role in pathogenesis of allergic airways disease partly through mediating phosphorylation of STAT3, thus increasing IL-1β–induced proinflammatory signalling (177). In addition, the nuclear PKM2-STAT3 pathway is implicated in LPS-induced lung injury (178). These findings indicate that PKM2 exhibits a proinflammatory effect by phosphorylating STAT3. The PKM2 activator, TEPP-46 exerts an anti-inflammatory effect and low activation of STAT3 (177).

Therefore, PKM2 promotes the production of proinflammatory cytokines, and expression of PKM2 significantly promotes inflammatory responses. Shikonin and other PKM2 inhibitors exert an anti-inflammatory effect by preventing the production of proinflammatory cytokines. Hence, the design of drugs targeting PKM2 inhibition can reduce the release of pro-inflammatory cytokines and alleviate cytokines storms.

### 5.2 Bidirectional Role of PKM2 in Maintenance of Cellular Redox Homeostasis

Oxidative stress is an imbalance between production of free radicals and ROS, and their elimination by protective mechanisms, such as antioxidants. This imbalance can lead to the damage of important biomolecules and organs, with potential effects on the whole organism (179). Several studies have explored the mechanism through which continued oxidative stress can lead to chronic inflammation and further cause chronic diseases. Oxidative stress can activate various transcription factors such as NF-κB, HIF-1α, β-catenin and Nuclear factor E2-related factor (Nrf-2), resulting in the expression of over 500 different genes, including inflammatory cytokines and chemokines (180). Therefore, oxidative stress and inflammation are closely linked.

Some cells highly depend on PKM2 whereas some do not, therefore, their regulation of redox state through PKM2 modulation occurs through opposite mechanisms. Redox-regulated PKM2 directly binds to p53 and the redox status of cysteine-423 of PKM2 tetramer is important for differential regulation of p53 transcriptional activity. The PKM2 tetramer suppresses p53 transcriptional activity and apoptosis in a high oxidation state but enhances these two processes in a low oxidation state (181). TEPP-46 alleviates oxidative stress in cardiomyocytes and suppresses apoptosis of cardiomyocytes, thus preventing cardiac dysfunction, whereas it exacerbates oxidative stress and promotes apoptosis in lung cancer cells. These findings indicate the redox-dependent differences expressed in different tissues. In endothelial cells, cancer cells and neuronal cells, cellular metabolism is highly dependent on PKM2 expression and it’s mediated by aerobic glycolysis. Therefore, expression of PKM2 plays a protective role against oxidative stress in these cells. Studies have reported that PKM2 is a crucial neuroprotective target against oxidative stress (182). PKM2 drives GSH biosynthesis which is an antioxidant intermediate, thus protecting neurons from oxidative damage and conferring neuroprotective effects. In endothelial cells, active endothelial NO synthase (eNOS) interacts with PKM2 and S-nitrosates PKM2, thus reducing PKM2 activity. PKM2 inhibition increases substrate flux through the pentose phosphate pathway to generate reducing equivalents, including NADPH and GSH, thus protecting cells against oxidative stress (183). Moreover, PKM2 translocates to mitochondria under oxidative stress (184). In the mitochondria, PKM2 interacts with Bcl2 and phosphorylates it at threonine (T) 69, thus preventing Cul3-RBX1 ligase-mediated degradation of Bcl2, ultimately enhancing apoptosis resistance of tumour cells. ROS inhibits PKM2 activity thus contributes to cellular antioxidant responses (34). The inhibition of PKM2 activity diverts glucose flux into PPP to generate sufficient reducing potential for detoxification of ROS. PKM2 expression supports the survival of cancers under acute oxidative stress by promoting anti-oxidant responses, whereas PKM2 activators compromise both pro-anabolic and anti-oxidant functions of cancer cells by increasing PKM2 activity thus interfering with its metabolism. 3,3′,5-triiodothyroxine (T3), a hormone secreted from the thyroid gland protects cells by improving the redox state and regulates the expression of PKM (185). T3 can inhibit apoptosis and oxidative stress in human myocardial cells by upregulating the PKM2/PKM1 ratio. In addition, Nrf-2 regulates expression of several genes responsible for cellular detoxification, antioxidant function, anti-inflammatory effect, drug/xenobiotic transportation, and stress-related factors (186). Astrocytic DRD2 induces GSH synthesis through PKM2-mediated Nrf2 transactivation (187).

PKM2 expression plays different roles in oxidative stress in cells of different tissues. Selective protection against oxidative damage can be achieved by activating or inhibiting PKM2 activity. Although inhibition of PKM2 can significantly alleviate inflammation, complete knockdown of PKM2 is not feasible as it causes damage to cells that are highly dependent on PKM2, such as endothelial cells. Therefore, selective modulation of PKM2 is important to reduce the secretion of inflammatory cytokines and suppression of cytokine storm through alleviation of oxidative stress.

### 5.3 Alleviating Inflammatory Damage by Pharmacologically Targeting PKM2

PKM2 forms a bridge between immunometabolic reprogramming and inflammatory dysfunction, therefore, PKM2 is a potential therapeutic target for the treatment of inflammatory disease. Several agents have been reported that improve inflammatory damage by inhibiting PKM2 expression. Increased glucose uptake and glycolytic flux promotes generation of mitochondrial ROS in monocytes and macrophages of patients with atherosclerotic coronary artery disease (CAD), which in turn promotes PKM2 dimerization thus inducing its nuclear translocation (9). Nuclear PKM2 functions as a protein kinase and phosphorylates STAT3 and further promotes the production of IL-6 and IL-1β, resulting in systemic and tissue inflammation. PKM2 serves as a molecular integrator of metabolic dysfunction, oxidative stress, and tissue inflammation, representing a novel therapeutic target for treatment of cardiovascular disease and other inflammatory diseases. Notably, PKM2-mediated glycolysis is significantly increased in the complete Freund’s adjuvant-induced astrocyte activation, and platelet-rich plasma effectively inhibits inflammatory response and HMGB1 expression by regulating PKM2-mediated glycolysis and STAT3 signalling-mediated astrocyte activation (188). PKM2-mediated aerobic glycolysis and protein kinase interactions play important roles in cytokine production and inflammation and provide a basis for the development of PKM2-targeted anti-inflammatory drugs.

Activators of PKM2 activity and PKM2 dimer inhibitors can suppress cytokine production and abrogate inflammatory diseases by mediating immunometabolic reprogramming. In addition, several pharmacological active ingredients have been reported that target PKM2 to modulate inflammatory responses. Plumbagin (5-hydroxy-2-methyl-1, 4-naphthoquinone) is a quinone isolated from the roots of *Plumbago zeylanica*. Previous studies reported that plumbagin exerts an anti-inflammatory effect by regulating pro-inflammatory signalling (189–191). Plumbagin was shown to protect mice from lethal endotoxemia and polymicrobial sepsis induced by cecal ligation and puncture (CLP) by inhibiting the NADPH oxidase 4 (NOX4)/PKM2-dependent immunometabolism pathway, which inhibits LPS-induced PKM2 expression, lactate production and subsequent proinflammatory cytokine (IL-1β and HMGB1) release in macrophages (192). These findings indicate that plumbagin downregulates PKM2 expression to control proinflammatory cytokine production. The traditional Chinese medicine formula Xijiao Dihuang decoction (XJDHT) was first reported in the medical classic “Beiji Qianjin Yaofang” and TCM doctors utilize XJDHT to improve prognosis of patients with sepsis. The main components of XJDHT include Rehmannia, Peony, Cortex Moudan and Cornu Bubali. Studies have shown that XJDHT improves survival of rats with sepsis by modulating the HIF-1α signalling pathway and inhibiting the release of inflammatory cytokine, such as IL-6 (193). Recent studies reported that XJDHT alleviates sepsis by reducing the release of proinflammatory cytokines. XJDHT can improve sepsis by suppressing aerobic glycolysis through downregulation of the TLR4/HIF-1α/PKM2 signalling pathway (194). Therefore, the treatment effect of XJDHT on sepsis indicates that cytokine release can be inhibited by modulating PKM2. However, some agents reduce ROS production and alleviate oxidative stress by upregulating PKM2 in PKM2-dependent cells such as endothelial cells. Qiliqiangxin is a traditional Chinese medicine preparation, comprising 11 Chinese herbal medicines, including astragalus, ginseng and aconite. A multicentre, randomized, double-blind, parallel-group, placebo-controlled study reported effective treatment of 512 chronic heart failure patients using qiliqiangxin (195). The therapeutic effect of qiliqiangxin is through improving cardiomyocyte metabolism and inhibiting cardiomyocyte apoptosis (196–199). Qiliqiangxin improves glucose utilization and metabolism and increases ATP production by upregulating HIF-1α and several glycolysis-related enzymes, including PKM2, thus protecting cardiac microvascular endothelial cells against hypoxia injury (15). In addition, the effects of Chinese medicine in controlling macrophage polarization by modulating PKM2 have been reported. Lycium barbarum polysaccharide is the main bioactive component of Chinese wolfberry, and this compound inhibits PKM2 ubiquitination by upregulating the expression of ubiquitin ligases, thus suppressing LPS-induced inflammation by modulating glycolysis and M1 macrophage polarization (200). Therefore, targeting PKM2 to inhibit glycolysis in an overactive macrophage can provide a new therapeutic option for the treatment of inflammatory diseases.

Several drugs that inhibit cytokine release and improve inflammatory damage by regulating PKM2 expression have been reported. However, effective pharmacological interventions for effective treatment of cytokine storms have not been designed. Therefore, drugs that target and modulate PKM2 to inhibit inflammatory storms should be designed.

## 6 Protective Role of PKM2 in Specific Cells

PKM2 is highly expressed in rapidly dividing cells whereas its PK activity is significantly decreased. Notably, inhibition of PKM2 by cell growth signals can uncouple the ability of cells to assimilate nutrients into biosynthetic pathways from production of ATP (12). Therefore, PKM2 is an important regulator of cancer cell metabolism, and inhibition of PKM2 can block cancer cell proliferation and excessive immune cell responses (2). However, endothelial cells and a few other types of cells display similar rapid proliferation characteristics such as cancer cells. Therefore, metabolism is a key regulator of growth of endothelial cells, in particular angiogenesis (201, 202). Glycolysis is a driving force for endothelial cell proliferation, whereas the Warburg effect has been reported in endothelial cells (203). Studies have shown that endothelial cells express PKM2 almost exclusively over PKM1, which is significant for maintaining their tight junctions and barrier function (204). PKM2 is required for the suppression of p53 and for maintaining cell cycle progression in proliferating endothelial cells. On the contrary, PKM2 regulates vascular barrier function by suppressing NF-κB and its downstream target, the vascular permeability factor angiopoietin 2 in quiescent endothelial cells. Notably, the loss of PKM2 in endothelial cells results in TCA cycle dysfunction, changes in mitochondrial substrate utilization, thus impairing endothelial cell proliferation and migration (205). In addition, the loss of PKM2 can impair S-adenosylmethionine (SAM) synthesis, causing DNA hypomethylation, de-repression of endogenous retroviral elements and autoimmune activation. Moreover, infiltrated/activated neutrophils at wound site release PKM2 during early stages of wound repair, which facilitates early wound healing by promoting angiogenesis at the wound site (206). Furthermore, PKM2 activates the STAT3 pathway which in turn promotes tissue repair and functional recovery by enhancing neuroblast migration, neurogenesis, and angiogenesis after stroke (207). PKM2 has been proposed as a new myokine, which contributes to axonal extension and functional motor recovery in spinal cord injured mice (208). PKM2 targets valosin-containing protein (VCP) to increase the density of axons and motor function which are mediated by extracellular PKM2-VCP-driven ATPase activity (209). The expression of PKM2 dimer plays an important role in repairing injury by promoting endothelial cell proliferation.

The potential effects of systemic pharmaceutical PKM2 inhibition on normal cells have not been fully elucidated, thus potential on-target side effects of PKM2 modulation should be explored. Targeting PKM2 as an adjuvant antineoplastic therapy can damage the vascular barrier and increase permeability of the vascular endothelium, thus leading to sepsis in immuno-compromised patients. Therefore, it is necessary to be cautious when developing therapies against cancer or inflammation that target PKM2.

PKM2 exerts an antioxidant effect thus protecting PKM2-dependent cells, such as endothelial cells, from oxidative stress. This protective effect of PKM2 on endothelial alleviates kidney injury (210). Endothelial nitric oxide synthase (eNOS) plays a protective role against kidney injury. S-nitroso-CoA (SNO-CoA)–aldoketo reductase family member (AKR1A1) is highly expressed in renal proximal tubules, where it promotes the activity of eNOS in reprogramming intermediary metabolism which is regulated by PKM2, thus protecting kidneys against acute kidney injury. In addition, podocyte-specific PKM2-knockdown mice with diabetes present with worse albuminuria and glomerular pathology. PKM2 activation protects against diabetic nephropathy by increasing glucose metabolic flux, inhibiting production of toxic glucose metabolites and by inducing mitochondrial biogenesis to restore mitochondrial function (211). Therefore, PKM2 protects organ injury by abrogating oxidative stress in specific cells. Specificity of agents targeting PKM2 should be explored to avoid adverse side effects, however, PKM2 is an attractive target.

## 7 Conclusion

The cytokine storm is an overreaction of immune system, which excessively activates immune cells, resulting in secretion and release of high levels of proinflammatory cytokines ultimately leading to lethal inflammation. Although the cytokine storm is the leading cause of death in many inflammatory diseases, effective therapy options for treating cytokine storm storms have not been fully explored. A previous study reported that immune cells, but not cancer cells, dominate glucose metabolism in the tumour microenvironment, thus providing anew understanding of the tumour microenvironment and further indicated the importance of immunometabolic reprogramming (212, 213).

PKM2 is a pyruvate kinase with lower enzymatic activity compared with PKM1. PKM2 can promote inflammation by switching glucose metabolic pathways to glycolysis. Therefore, PKM2 is a crucial mediator between inflammation and metabolic disorder. The current study reviewed the role of PKM2-mediated immunometabolic reprogramming in cytokine storm through multiple pathways. Firstly, it was shown that PKM2-mediated Warburg effect activates immune cells and induces pro-inflammatory activity. Secondly, PKM2 directly promotes the secretion of cytokines by inducing metabolic reprogramming in cells, which results in cytokine storm. Finally, PKM2 was implicated in novel forms of pro-inflammatory cell death including pyroptosis and ferroptosis. PKM2-mediated activation of inflammasome and stress granule signalling triggers pyroptosis of cells. In addition, PKM2-dependent glycolysis can indirectly induce ferroptosis. However, the specific mechanisms on how PKM2 modulates pyroptosis or ferroptosis have not been fully elucidated.

Several studies have explored therapeutic methods and drugs targeting PKM2. These findings indicate that PKM2 represents a novel potential target for the development of anti-inflammatory drugs that can inhibit cytokine storm and for treatment of severe inflammatory diseases. However, the potential side effects of PKM2 modulation have not been explored.

Intensive studies and clinical trial should be conducted to validate the effectiveness of targeting PKM2 for treatment of inflammatory diseases. Therefore, the current study states some basics to be explored thus providing a guidance for further research. PKM2 exists in different isoforms in different cells of different species, and their intracellular localization differs across cells (9, 10, 77, 214). The exact roles that PKM2 plays in different cells and the mechanism of action has not been fully elucidated. In addition, the inhibition of PKM2 or activation of PKM2 tetramer is a double-edged sword in treatment of inflammatory disease, as PKM2 dominates metabolism in some cells such as endothelial cells (204). Studies should thus explore the possibility of specifically targeting PKM2 to prevent excessive immune response without injuring PKM2-depedent cells. Moreover, inflammatory responses facilitate innate immunity against infections, whereas overactivation of inflammatory cells leads to excessive inflammation resulting in sepsis and even death. Therefore, if inhibition of PKM2 and activation of PKM2 tetramer can prevent excessive immune response, however, this regulation can limit the function of immune system, thus leading to increase in pathogens in bodies. Therefore, studies should explore modulation of immune system by targeting PKM2, and elucidate if the therapy targeting PKM2 can be achieved in both infectious diseases and non-infectious inflammatory diseases. Further, transformation of PKM2 dimer and tetramer can be modulated by protein modifications such as phosphorylation, acetylation, and succinylation (32–34). Studies should explore if the structure and function of PKM2 can be modulated by designing agents that modify its protein modification processes. Addressing these issues is important in developing agents that target PKM2 for inflammatory diseases.

In summary, this review on PKM2 and immunometabolic reprogramming provide insights into the molecular and metabolic mechanisms resulting in cytokine storm and thus provide a basis for restoring balance in the immune system and for development of potential therapies for inflammatory diseases.

## Author Contributions

ZL, YL, and HC researched data and wrote the manuscript. JZ and DL established the scope of the review. All authors made substantial contributions to the discussion and revision of the manuscript before submission.

## Funding

The research was supported by the National Natural Science Foundation of China (No.82074079), the Natural Science Foundation of Zhejiang Province (No.LY19H280003), the Opening Project of Key Laboratory of Integrative Chinese and Western Medicine for the Diagnosis and Treatment of Circulatory Diseases of Zhejiang Province (No.2C32005), Zhejiang science and technology innovation program for college students (No.2021R410044) and “the Postgraduate Scientific Research Fund” of Zhejiang Chinese Medical University (No.2020YKJ15).

## Conflict of Interest

The authors declare that the research was conducted in the absence of any commercial or financial relationships that could be construed as a potential conflict of interest.

## Publisher’s Note

All claims expressed in this article are solely those of the authors and do not necessarily represent those of their affiliated organizations, or those of the publisher, the editors and the reviewers. Any product that may be evaluated in this article, or claim that may be made by its manufacturer, is not guaranteed or endorsed by the publisher.
